# Tracing CRISPR/Cas12a Mediated Genome Editing Events in Apple Using High-Throughput Genotyping by PCR Capillary Gel Electrophoresis

**DOI:** 10.3390/ijms222212611

**Published:** 2021-11-22

**Authors:** Susan Schröpfer, Henryk Flachowsky

**Affiliations:** Julius Kühn Institute (JKI)—Federal Research Centre for Cultivated Plants, Institute for Breeding Research on Fruit Crops, 01326 Dresden, Germany; henryk.flachowsky@julius-kuehn.de

**Keywords:** *Malus domestica*, Cpf1, *MdPDS*, fragment length analysis, amplicon deep sequencing, deletion, mutation

## Abstract

The use of the novel CRISPR/Cas12a system is advantageous, as it expands the possibilities for genome editing (GE) applications due to its different features compared to the commonly used CRISPR/Cas9 system. In this work, the CRISPR/Cas12a system was applied for the first time to apple to investigate its general usability for GE applications. Efficient guide RNAs targeting different exons of the endogenous reporter gene *MdPDS*, whose disruption leads to the albino phenotype, were pre-selected by in vitro cleavage assays. A construct was transferred to apple encoding for a CRISPR/Cas12a system that simultaneously targets two loci in *MdPDS*. Using fluorescent PCR capillary electrophoresis and amplicon deep sequencing, all identified GE events of regenerated albino shoots were characterized as deletions. Large deletions between the two neighboring target sites were not observed. Furthermore, a chimeric composition of regenerates and shoots that exhibited multiple GE events was observed frequently. By comparing both analytical methods, it was shown that fluorescent PCR capillary gel electrophoresis is a sensitive high-throughput genotyping method that allows accurate predictions of the size and proportion of indel mutations for multiple loci simultaneously. Especially for species exhibiting high frequencies of chimerism, it can be recommended as a cost-effective method for efficient selection of homohistont GE lines.

## 1. Introduction

The improvement of crop plants is an urgent necessity in order to respond to global problems such as climate change and the rapidly growing world population. In this context, the use and further development of innovative breeding technologies that enable efficient and targeted plant breeding is of great interest. Since the discovery and functional understanding of the first CRISPR/Cas (clustered regularly interspaced short palindromic repeats/CRISPR-associated protein) system in 2012 [1], a new molecular tool allowing precise genome editing (GE) in different kingdoms, including the plant kingdom [2,3], is now available. This tool acts as programmable RNA-guided DNA endonuclease [1] and allows the introduction of a DNA double-strand break (DSB) into a desired target locus, which is the starting point of genomic modifications. For the repair of DSBs, plants possess two major DNA repair mechanisms: non-homologous end-joining (NHEJ) and homology-directed repair (HDR) [4]. Repair by the error-prone NHEJ pathway is a popular technique for targeted mutagenesis of genes, as it is associated with small insertions and/or deletions (so-called indel mutations) at the site of the DSB [5]. HDR-mediated GE is dependent on a homologous repair template and can be used to introduce specific point mutations and to insert or replace sequences at genomic target loci.

Until now, two classes of CRISPR/Cas systems have been discovered, which were further divided into six subtypes. Class 1 CRISPR/Cas systems (subtypes I, III, IV) require multiple effector proteins; in class 2 systems (subtypes II, V, VI), only one single effector protein is required [6]. The most studied and widely used CRISPR/Cas9 system from *Streptococcus pyogenes* Rosenbach belongs to class 2 subtype II and consists of the *Sp*Cas9 effector, a CRISPR RNA (crRNA) and a trans-activating CRISPR RNA (tracrRNA) [7]. For use in GE applications, both RNA components can be fused to each other and used as a single guide RNA (sgRNA) [1]. A further CRISPR nuclease suitable for GE applications is Cas12a, previously named Cpf1 (CRISPR from *Prevotella* and *Francisella* 1), which belongs to class 2 subtype V of CRISPR systems [8]. Different orthologs of Cas12a were isolated from *Francisella novicida* U112 (*Fn*Cas12a), *Acidaminococcus* spp. BV3L6 (*As*Cas12a), and *Lachnospiraceae bacterium* ND2006 (*Lb*Cas12a) and assessed for GE in eukaryotes [9,10]. Furthermore, several studies have already demonstrated the potential of Cas12a for GE in model plants such as in *Nicotiana benthamiana* Domin, *Solanum lycopersicum* L., *Arabidopsis thaliana* (L.) Heynh. [11], and also in crops like *Citrus ×paradisi* Macfad., *Glycine max* (L.) Merr., and *Oryza sativa* L. [12,13,14]. The CRISPR/Cas12a system differs from CRISPR/Cas9 in some structural and mechanistic properties, thus extending and improving possibilities for GE applications.

The first study on GE in temperate fruit trees using CRISPR/Cas9 was reported in 2016, when this system was used for targeted mutagenesis of the endogenous *phytoene desaturase gene* (*PDS*) of *Malus domestica* Borkh [15]. As *PDS* is an essential gene in carotenoid and chlorophyll biosynthesis pathways and its disruption leads to albino and dwarf phenotypes [16], it is widely used as a reporter gene for initial GE studies in plant species. The modification of traits by targeted mutagenesis of apple genes using the CRISPR/Cas9 system was also demonstrated with the aim of reducing susceptibility to fire blight disease [17], to induce early flowering [18], and to improve resistance to ring rot [19]. Other GE studies on apple using the CRISPR/Cas9 system were aimed at technical extensions and advances such as the delivery of CRISPR/Cas9 ribonucleoproteins (RNPs) for DNA-free GE in apple protoplast [20,21], minimizing the trace of exogenous DNA by inducing a FLP/recombination system [17], reducing the initial chimerism of genome edited tissue [22], or the use of modified Cas9 enzymes for precise base editing (BE) [22]. Recently, the application of the CRISPR/Cas9 technology to wild apple *Malus sieversii* (Ledeb.) Roem. was shown [23]. To our knowledge, there have been no studies on the use of CRISPR/Cas12a in apple to date.

The screening and identification of mutations on target sites is a critical step in the workflow for the establishment of GE plants. Especially for GE applications in apple, a powerful detection method is necessary, as complex GE profiles and chimerism were frequently observed [18]. In general, several methods have been developed for detection of CRISPR/Cas induced mutations, which differ in their informative value about the type of mutations, their sensitivity, the possibility of use at a high-throughput scale, and the investment of time and costs [24]. The mismatch cleavage assay is frequently used and based on enzymatic cleavage of heteroduplex DNA at mismatches or extrahelical loops formed by single or multiple nucleotides [25]. Other detection methods are also available, such as high-resolution melting curve analysis (HRMA) [26], heteroduplex mobility assay [27], analysis of cleaved amplified polymorphic sequences (CAPS) [28], loss of primer binding site [29], amplified fragment length polymorphism (AFLP) [30], restriction fragment length polymorphism (RFLP) [31], fluorescent PCR capillary gel electrophoresis [32], cloning and Sanger sequencing [33], and amplicon deep sequencing [34]. The benefit from methods based on sequencing is the generation of detailed information about the concrete mutation on the DNA sequence level [24]. Moreover, amplicon deep sequencing is highly informative for the calculation of mutation frequencies with a high sensitivity [35].

The results presented herein focused on the use of the CRISPR nuclease *Lb*Cas12a, demonstrating its suitability as novel tool for GE in apple. Moreover, comparing different methods for detection and characterization of mutations, fluorescent PCR capillary gel electrophoresis was shown to be a powerful high-throughput methodology that allows the precise prediction of the size and proportion of indel mutations of multiple loci in chimeric tissue in a single reaction.

## 2. Results

### 2.1. Design and Pre-Selection of Efficient MdPDS Specific crRNAs for LbCas12a

**Figure 1:**
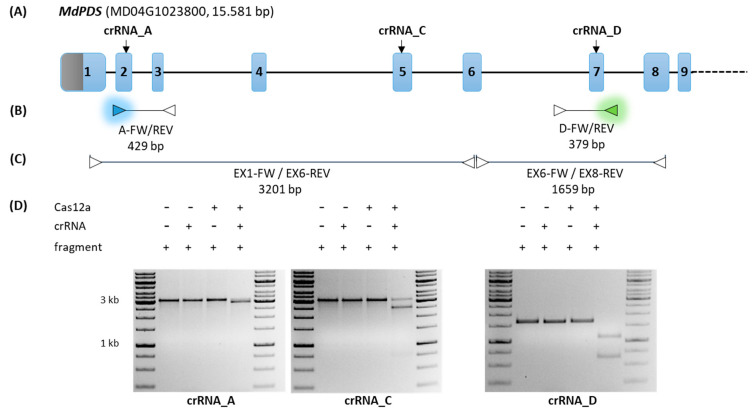
The *MdPDS* target locus for GE analysis. (**A**) The scheme represents the genomic organization of *MdPDS* (MD04G1023800) comprising exons one to nine (numbered boxes; 5’-UTR (gray), coding sequence (blue)) and introns (black line). Target sites of crRNAs in coding exon 2 (crRNA_A), exon 5 (crRNA_C), and exon 7 (crRNA_D) are indicated by arrows. (**B**,**C**) Binding sites of primer pairs used for fluorescent PCR capillary gel electrophoresis (**B**) and DNA cleavage assays (**C**) are represented as triangles. Primers labeled with fluorescent dyes are represented in blue and green. (**D**) For DNA cleavage assays, PCR fragments were amplified from genomic DNA of ‘Gala’ and used as substrates for digestion with pre-assembled *Lb*Cas12a/crRNA complexes. Experimental controls without the *Lb*Cas12a/crRNA complex or with only one complex component each were also performed (indicated by + and −). The resulting products were separated by gel electrophoresis and, dependent on the target site of the respective crRNA, the cleavage of the PCR fragment resulted in different products: crRNA_A (fragment EX1-FW/EX6-REV: 2981 bp + 220 bp); crRNA_C (fragment EX1-FW/EX6-REV: 2576 bp + 626 bp); crRNA_D (fragment EX6-FW/EX8-REV: 1031 bp + 628 bp).

*MdPDS* represents a suitable reporter gene for in vivo GE experiments, as the knock-out of this gene leads to albino tissue [15]. Three different *MdPDS* specific crRNAs for *Lb*Cas12a (named crRNA_A, crRNA_C, and crRNA_D) were designed and selected with the help of the CRISPR RGEN Tools software targeting exons two, five, and seven, respectively (Figure 1A). Binding sites of primer pairs used in this study and resulting *MdPDS* specific fragments are represented in Figure 1B,C. The target loci in *MdPDS* are hereafter referred to as locus A, locus C, and locus D. The selected crRNAs have no potential off-target sites in the genome, as predicted by the software using the *Malus domestica* genome v3.0 as reference.

To test the functionality and assess the cleavage efficiency of these guide RNAs prior to in vivo experiments, in vitro DNA cleavage assays were performed. DNA substrates containing the target sequence (Figure 1C) were digested by *Lb*Cas12a that was pre-assembled with the respective crRNA. DNA cleavage assays using DNA substrates amplified by PCR from genomic DNA of the apple cultivar ‘Gala’ are presented in Figure 1D. *Lb*Cas12a assembled with crRNA_A or crRNA_D exhibit highly efficient nucleolytic activity, as the DNA substrates were fully digested. Only a partial digestion could be detected using crRNA_C, which indicates a lower cleavage efficiency on this specific DNA substrate originating from ‘Gala’. Additionally, DNA cleavage assays were also performed with DNA substrates amplified from the cultivars ‘Pinova’, ‘Golden Delicious’ and ‘Braeburn’ (Figure S1). In these experiments, a high in vitro cleavage efficiency could be observed for all tested crRNAs (crRNA_A, crRNA_C, and crRNA_D). The lower cleavage efficiency of crRNA_C on ‘Gala’ DNA may be due to DNA sequence differences between the different apple genotypes.

However, for subsequent in vivo experiments using ‘Gala’, crRNA_A and crRNA_D were selected, for which a high cleavage efficiency was demonstrated in vitro.

### 2.2. MdPDS Mutagenesis by T-DNA Transformation of CRISPR/LbCas12a Construct

**Figure 2:**
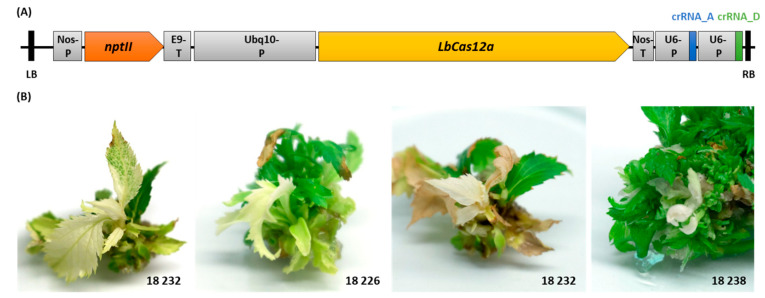
Transformation with the *CRISPR/LbCas12a* construct targeting *MdPDS*. (**A**) The T-DNA region of the binary plasmid p9oN-U10LbCpf1-Ex27 used for transformation of ‘Gala’ is shown. The T-DNA was flanked by left and right border sequences (LB and RB) and contained a *neomycin phosphotransferase II* (*nptII*) resistance cassette optimized for gene expression in plants that was under the control of the promoter from the *nopaline synthase gene* (Nos-P) and the *rbcS-E9* gene terminator (E9-T). The promoter of the *polyubiquitin 10* (*UBQ10*) gene from *A. thaliana* (Ubq10-P) regulated the expression of the *LbCas12a* gene. Downstream of the *LbCas12a* gene is the terminator from the *nopaline synthase gene* (Nos-T). The expression of two crRNAs targeting the *MdPDS* loci A (exon 2) and D (exon 7) was driven by one *A. thaliana U6* promoter (U6-P) each. (**B**) Examples of different regenerates resulting from T-DNA transformation with the *CRISPR/LbCas12a* construct containing shoots with albino tissue are shown.

A *CRISPR/LbCas12a* T-DNA construct (Figure 2A) was assembled, which consists of expression cassettes for *LbCas12a* and the two crRNAs: A and D. Furthermore, an *nptII* resistance cassette for the selection of transformed plant tissue was located within the construct. By *Agrobacterium tumefaciens*-mediated T-DNA transformation, the *CRISPR/LbCas12a* construct was transferred into the genotype ‘Gala’. Three independent transformation experiments were performed with a total of 660 leaves used for co-culture. Out of these, 1570 leaf explants could be obtained, which were used for regeneration on kanamycin containing selective media. For further in vitro cultivation, 163 regenerates were harvested.

A series of regenerates exhibited tissue or shoots with white color (Figure 2B), which was expected, as the knock-out of *MdPDS* should lead to the albino phenotype [15]. For a subset of 23 in vitro shoots with the albino phenotype, the transgenic state was analyzed by *nptII* and *LbCas12a* specific PCRs eleven months after transformation. For 19 shoots, both transgenic elements could be detected, whereas four shoots contained neither *nptII* nor *LbCas12a* specific sequences. The suitability of each DNA sample as a template for PCR amplification was shown using a universal primer pair for the amplification of an endogenous apple gene *MdEF1α*. A reliable discrimination between kanamycin-mediated chlorosis of non-transgenic tissue and the albino phenotype potentially due to mutagenesis of *MdPDS* could not be made. Nevertheless, the albino phenotype served as a marker to preselect shoots that were subsequently investigated for potential mutagenesis of *MdPDS* by molecular methods. After continuous selection on kanamycin containing medium for a total of 30 months, 18 regenerates (11% of the total) could be obtained that possessed large stretches of albino tissue.

### 2.3. High-Throughput Genotyping Indels Using Fluorescent PCR Combined with Capillary Gel Electrophoresis

**Figure 3:**
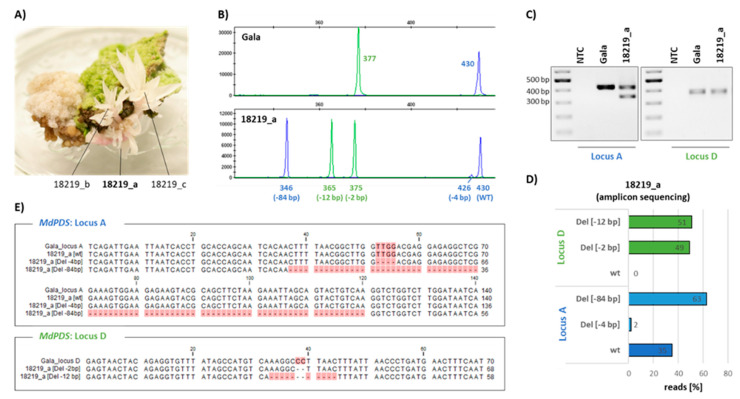
Molecular characterization of albino in vitro shoots by fluorescent PCR capillary electrophoresis and amplicon deep sequencing. (**A**) Single regenerated shoots exhibiting the albino phenotype were used to analyze GE events in target loci A and D within the *MdPDS* gene. (**B**) The *MdPDS* target loci were amplified by PCR using specific primer pairs labeled with fluorescent dyes (locus A: 6-FAM (blue); locus D: Atto532 (green)). The fragment lengths of the PCR products were determined by capillary gel electrophoresis. The resulting chromatograms (x-axis: fragment size; y-axis: fluorescence intensity (RFU)) are displayed for the non-transgenic wild type ‘Gala’ and the transgenic shoot 18219_a. The fragment lengths, as well as their deviation compared to the wild type alleles, are indicated. (**C**,**D**) PCR products flanking loci A and D were amplified from the identical template DNA with the same, but unlabeled, primer pair and analyzed by amplicon deep sequencing (NTC: no template control). The abundance of unique sequences and their difference in length compared to the reference sequence of ‘Gala’ was calculated (Del: deletion; wt: wild type). The diagram represents the percentage of total reads of the unique sequences. (**E**) The obtained unique sequences were aligned to the reference sequence of wt ‘Gala’. Differences from the consensus sequence are highlighted.

As shown in other studies [15,18], indels were expected as the most prominent GE events after the induction of DBSs in apple. To genotype albino shoots (Figure 3A) for indels, a method using fluorescent PCR combined with capillary gel electrophoresis was established (Figure 3B).

For this purpose, two differentially fluorescent-labeled primer pairs were designed, flanking the *MdPDS* target loci A (blue) and D (green), respectively (Figure 1B). These were used in a multiplex PCR to amplify both loci in a single reaction (Figure 3B). To allow the detection and discrimination of large deletion events between loci A and D (~ deletion of 4 kb), possibly amplified by the primer pair A-FW/D-REV, these two primers were used for fluorescent labeling. This resulted in the dual labeling of such PCR products. Amplified PCR fragments were separated by capillary gel electrophoresis and fragment length analysis was done relative to the internal fragment size standard. 

In the chromatogram of wild type (wt) ‘Gala’ shown in Figure 3B, the blue peak with a measured size of 430 bp represents the PCR product amplified from locus A and the green peak (377 bp) belongs to locus D. In comparison, the chromatogram of a selected albino shoot (transgenic regenerate 18 219, Figure 3A) contained peaks with different fragments. For locus D, this line possessed two fragments with similar peak sizes of 365 bp and 375 bp, which were 12 bp and 2 bp smaller, respectively, than that of the wt. This suggests a bi-allelic mutation at locus D. The amplification of locus A resulted in three fragments with sizes of 346 bp (84 bp smaller than wt), 426 bp (4 bp smaller than wt), and 430 bp (similar to wt). It is remarkable that peak height was different, especially for the fragment at 426 bp. Since more than two alleles of locus A were found in the diploid shoot, the analyzed tissue was probably chimeric.

From a total of 114 regenerates obtained within eleven months after transformation, 41 (36%) exhibited albino shoots. These shoots were further analyzed by fluorescent PCR capillary gel electrophoresis. Nine of them (8% from total) contained genome edited alleles at both loci. GE of only one single locus was not observed.

To verify results from fluorescent PCR capillary gel electrophoresis, indicating the presence of genome edited alleles for multiple target loci in a single reaction, the GE events were additionally characterized by amplicon deep sequencing.

### 2.4. Verification of GE Events by Amplicon Deep Sequencing

To characterize potential GE events of albino shoots on the sequence level, deep sequencing of PCR products amplified from loci A and D (Figure 3C) was performed. The type of GE event, such as deletion, insertion, or substitution, was determined by comparing the obtained unique sequences with the reference sequence obtained from wt ‘Gala’ (Figure 3D,E).

Amplicon deep sequencing of locus D of the albino shoot 18219_a resulted in a total of 167,120 reads analyzed (Table 1). Half (51%) of these reads corresponded to a sequence that has a deletion of 12 bp and the other half (49%) were attributable to a sequence that has a deletion of 2 bp (Figure 3D,E). For locus A, 35% of the reads could be assigned to the wt sequence, 63% to a sequence containing an 84 bp deletion, and 2% contained a deletion of 4 bp.

The deletion lengths of the GE events in 18219_a are consistent with the allele size differences observed by fragment length analysis. To proof this observation, 11 albino shoots that originated from five independent regenerates were investigated by the same methods. The results are summarized in Table 1. Each allele of locus A that was detected using fragment length analysis (in total, 30 measurements) could be confirmed by deep sequencing; for locus D, 38 from 41 alleles matched. Only a few, single sequences of GE events detected by sequencing were not identified using fragment length analysis. However, the proportion of reads of these sequences was always low. Using the primers specific for locus A, two artefacts were amplified from regenerate 18 298. These two sequences, called Art-165 bp and Art-172 bp (Table 1), contained the flanking primer sequences, but the inner sequence of the amplicons did not align to the target locus.

Besides the coherence regarding the indel size, a second correlation was observed between the percentage of reads from deep sequencing and the percentage of total peak height (Table 1), which expresses the relative fluorescence intensity of the measured PCR fragments. Correlation coefficients of 0.9928 for locus A and 0.9772 for locus D were calculated. The same calculation was performed using peak area as the parameter, which resulted in equivalent correlation coefficients (locus A: r = 0.9901; locus D: r = 0.9766).

Overall, these comparisons between the two independent methods clearly showed that fluorescent PCR capillary gel electrophoresis produced robust results for genotyping and that the size differences from wt alleles and the percentage of the total peak height can be used to predict the type and proportion of GE events in the analyzed tissue.

### 2.5. Characterization of GE Events in Apple, Introduced by CRISPR/LbCas12a 

**Figure 4:**
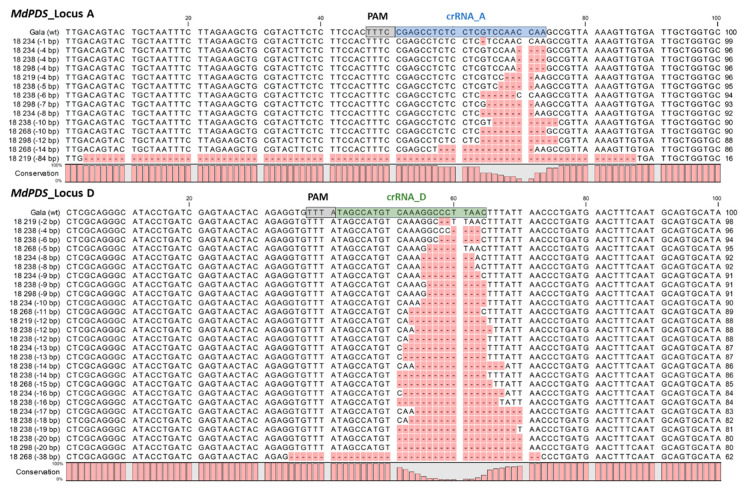
GE events in the apple *MdPDS* gene introduced by *Lb*Cas12a. The genomic loci (*MdPDS*_locus A and D) of different apple regenerates transformed with the CRISPR/*Lb*Cas12a construct were sequenced by amplicon deep sequencing. All resulting unique sequences containing GE events were aligned to the reference sequence from wt ‘Gala’ and sorted by deletion length in ascending order. The protospacer adjacent motif (PAM) and the target sites of the crRNAs A (blue) and B (green) are indicated in the wt sequence.

The sequences of loci A and D that resulted from independent GE events (different regenerates) are represented in the alignments shown in Figure 4. All GE events detected contain a deletion that is located in the target region of the respective CRISPR/*Lb*Cas12a nuclease complex. No insertion of sequences at the target site was found. The deletion length of 14 different GE events of locus A ranged between 1 and 84 bp, with an average of 12.4 bp. The 27 different GE events of locus D contained deletions of 2 to 38 bp, with an average of 13.0 bp. A large deletion event between both loci, which are ~4 kb distant from each other (Figure 1), was not detected.

### 2.6. Tracing GE Events in Chimeric Regenerates 

The results summarized in Table 1 show that several albino shoots contained a series of different alleles either at one locus (A or D) or both loci. For example, four different alleles of locus A and seven different alleles of locus D could be detected in the albino shoot of regenerate 18 234. These results indicated a chimeric origin of the analyzed tissue. 

**Figure 5:**
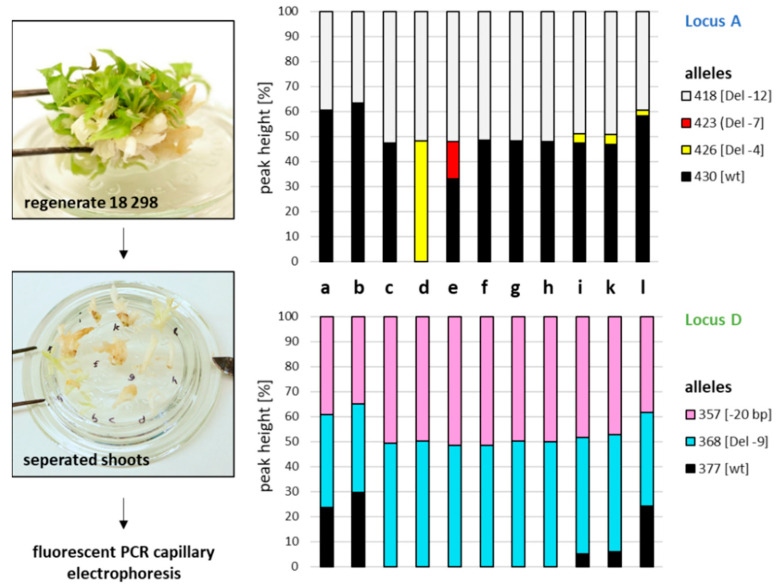
Distribution of CRISPR/*Lb*Cas12a induced GE events in a chimeric regenerate. Different albino shoots originating from tissue transformed with the *CRISPR/LbCas12a* construct were separated and genotyped by fluorescent PCR capillary gel electrophoresis. The percentages of the total peak heights of each allele detected for locus A (upper panel) and locus D (lower panel) are represented in the diagrams.

Using fluorescent PCR capillary gel electrophoresis, the type and the proportion of wt and genome edited alleles were investigated in single shoots of one regenerate (Figure 5). For the wt ‘Gala’, only one allele could be detected for each locus (corresponds to 100% of total peak height; Table 1), indicating a homozygous state of the respective allele in the diploid genotype. All shoots of regenerate 18 298 (Figure 4, upper diagram) contained the same allele of 418 bp for locus A, corresponding to a deletion of 12 bp. This allele occurred together with the wt allele in ten out of the eleven shoots. This indicated that this GE event must have occurred very early after transformation and was stably disseminated during the regeneration phase. Subsequent GE events of the remaining wt allele occurred independently in different cells of the regenerate, leading to the alleles 423 and 426. Allele 426 (deletion of four bp) was found with only a small proportion in three chimeric shoots. One non-chimeric GE shoot (d) with a bi-allelic mutation of locus A was detected. The peak height of each of the two alleles 418 and 426 was ~50% of the total peak height in shoot d. 

For locus D, the two GE alleles 357 and 368 were detected in each shoot investigated, suggesting an early and efficient GE of locus D on both chromosomal arms. In five out of eleven shoots, the wt allele could additionally be detected, indicating chimeric tissue. For the other six shoots (c–h), a bi-allelic mutation of locus D was demonstrated.

In the present study, a genome edited apple mutant (18298_d) could be identified, which contained bi-allelic mutations at two independent loci within the *MdPDS* gene. To identify which of the *MdPDS* mutations at locus A and D occur together on one chromosomal arm, the whole segment of ~4.3 kb was amplified, cloned, and analyzed by Sanger sequencing. A common occurrence of allele 418 (−12 bp deletion) of locus A and allele 368 (−9 bp deletion) of locus B was identified in five clones. Two clones contained allele 426 (−4 bp deletion) of locus A and allele 357 (−20 bp deletion) of locus D. Based on the proportions of the alleles in the examined tissue, which is ~50% for each allele (Figure 5), it can be concluded that the mutant 18298_d is genotypically uniform and not chimeric. 

## 3. Discussion

In this study, the CRISPR/Cas12a system was applied for the first time to apple, successfully demonstrating targeted mutagenesis of multiple loci in genome-edited apple shoots. As a result, the CRISPR/Cas12a system can be added to the toolbox for GE of apple, broadening its spectrum of potential applications due to the distinct features of Cas12a compared to Cas9. Different from Cas9, for which a G-rich protospacer adjacent motif (PAM) is preferred [36], Cas12a uses the T-rich PAM sequence 5′-TTTV-3′ (V is A, G, or C) for target DNA recognition [8]. This feature expands possible target sequences in the genome, e.g., for the targeting of AT-rich sequences characteristic for plant promotor regions or introns [10,37]. Unique to Cas12a is its cleavage activity, leading to staggered ends with 5–8 nt long 5’-overhangs, which were described to promote site-directed integration events [8]. Furthermore, the Cas12a cleavage site is located distal to the PAM [8], unlike that of Cas9, which cuts proximal to the PAM, producing blunt ends or 1 nt overhangs [1]. The cleavage position of Cas12a corresponds to the position of indel mutations observed in this study (Figure 4). The Cas12a-mediated cut, located far away from the PAM and seed region, is advantageous for targeted integration of DNA by in planta gene targeting (ipGT) and HDR-based applications [37]. The enhanced frequency of ipGT compared to Cas9 by the use of Cas12a in the plant model organism *A. thaliana*, has already demonstrated [37]. Additionally, Cas12a does not require a second tracrRNA molecule [8] and the mature crRNA of Cas12a is shorter than the sgRNA of Cas9. The shortness of the crRNAs of Cas12a, consisting of 42–44 nt, including a 23–25 nt long target specific guide sequence [8], allows the cost and time efficient generation of guide RNAs by chemical syntheses. This option facilitates, for example, the pre-selection of efficient crRNAs by in vitro cleavage assays prior to time consuming in vitro GE experiments, as was performed in this study (Figure 1 and Figure S1). Additionally, the shorter guide RNA along with the smaller molecular size of Cas12a will simplify DNA-free GE by the application of CRISPR/Cas12a RNPs in the future. However, the CRISPR/Cas12a system also has some limitations. These include the temperature sensitivity of the enzyme, exhibiting a reduced activity of the enzyme at ambient temperatures (e.g., 20–25 °C), which is mandatory for plant transformation and cultivation [11,38,39,40,41]. Recently, an optimized temperature-tolerant variant of *Lb*Cas12a (called ttLbCas12a) was engineered for high-efficient GE in plants [39]. Presumably, this Cas12a variant could increase GE efficiency in apple as well. In addition, the range of available Cas12a orthologs is expanding. Recently, six novel Cas12a orthologs were shown to have high editing activity in rice and to be suitable for large multiplex editing approaches [42].

As summarized by Nishitani et al. [43], the range of calculated mutation efficiency in previous GE studies on apple using *Sp*Cas9 is wide and varies from 3–93%. Despite differences in the experimental designs, different parameters were also used to calculate mutation efficiencies, e.g., phenotypic markers such as the albino phenotype caused by the mutation of *MdPDS* or the early flowering phenotype mediated by the knock-out of *MdTFL1.1.* In this study, the mutation efficiency based on the albino phenotype was not calculated, because this phenotype was shown not to be a reliable marker for GE of apple when used in combination with, e.g., kanamycin for selection of transformants. As the sensitivity of non-transformed tissue to kanamycin also results in chlorosis prior to death of the tissue [44,45], a clear discrimination of a kanamycin or *MdPDS-*mutation caused phenotype was not possible in our study. Using fluorescent PCR capillary gel electrophoresis, an indel mutation efficiency of 8% was observed in the performed transformation experiments. 

In general, it is known that DSB repair mechanisms differ from species to species and in some species, deletion events are the preferred repair outcome [46]. In previous studies using Cas9 for targeted mutagenesis of apple genes, deletions were observed as a main GE event at the site of the induced DSB [15,18,20]. The frequent occurrence of insertions was also reported, which seems to depend on the target sequence [20], whereas substitutions were reported only to a limited extent. In this study, using Cas12a for the simultaneous induction of DSBs in two neighboring target loci, only deletions with an average size of about 12 to 13 bp were observed, which is comparable to Cas9 mediated GE. Furthermore, simultaneous GE of both target sites was found in each genome-edited shoot, but large deletions between the two target sites were not detected. This is consistent with the findings of Charrier et al. [18], who used Cas9 for targeting of two loci with a distance of 272 bp in apple. Therefore, large deletion events occur rather rarely or not at all in apple, which is different compared to other species like poplar, kiwifruit, or grape [47,48,49]. Furthermore, complex editing profiles and a high frequency of chimerism were observed in apple using Cas9 for GE [18]. Furthermore, in this work, multiple Cas12a-edited alleles from the same locus were detected in a single regenerate or shoot, which can be explained by a chimeric composition of the analyzed tissue. This is the result of timely and locally independent Cas12a-mediated somatic mutation events, which occur early or late after transformation in different cells of the same organism. The trace of individual GE events in chimeric regenerates was demonstrated in detail in this work (Figure 5), thus illustrating the genesis of chimerism in GE applications in apple. These analyses further emphasize the need to eliminate chimerism for the generation of genetically uniform, homohistont GE apple lines. As shown before, several rounds of in vitro regeneration and propagation are useful to achieve chimera separation in tree plants [50], which has already been successfully applied for mutagenesis approaches with Cas9 [22]. 

Fluorescent PCR capillary gel electrophoresis was used in this study for the detection of indels on CRISPR target sites. Multiple target sites can be analyzed in a single reaction, using different fluorescent dyes for primer-labeling. Two targets were analyzed simultaneously in this study, which can be expanded further. Using standard dye sets, up to four different targets sites can be analyzed in one reaction, which was already demonstrated [51]. The method was evaluated in this work by comparison of the obtained results with data from amplicon deep sequencing, which is the most sensitive and informative method with regards to the nature and frequency of mutations [24]. In respect to the detected indel sizes, a high accuracy to a single base was observed in a range of 1 to 84 bp for fluorescent PCR capillary gel electrophoresis. This allows precise characterization of GE events and thus, selection of desired frame-shift mutations to knock-out gene functions. Additionally, a high sensitivity was demonstrated, as indels with a low frequency of about 1–2% of the reads were detected. Furthermore, the proportion of detected alleles in the analyzed tissue can be calculated from observed peak heights, and a strong correlation was shown with the frequency of reads obtained by amplicon deep sequencing. This feature is helpful in analyzing potentially chimeric tissue, which was already addressed as an important point for GE applications. It should be mentioned, however, that fluorescent PCR capillary gel electrophoresis is limited to the detection of indels, which represent the main product of CRISPR-based mutagenesis in apple. Furthermore, it cannot be excluded that different mutations with the same indel size are present in the tissue that cannot be distinguished. This was shown for two different GE events, both resulting in a 12 bp deletion in shoot c of regenerate 18 238 (Table 1). Thus, final characterization of genome edited lines by sequencing based methods is necessary for this reason. Altogether, fluorescent PCR capillary gel electrophoresis represents a cost effective, sensitive high-throughput detection method for identification and characterization of indels, allowing conclusions regarding the sizes and frequency of observed GE events.

## 4. Materials and Methods

### 4.1. Oligonucleotides and Kits

All oligonucleotides used in this study as crRNA or DNA primers are listed in Table S1 and were ordered from biomers.net GmbH (Ulm, Germany). The DNeasy Plant Mini Kit (Qiagen GmbH, Hilden, Germany) was used for extraction of genomic DNA of apple tissue and the GeneJET Plasmid Miniprep Kit (Thermo Fisher Scientific Inc., Waltham, MA, USA) for isolation of plasmid DNA from bacterial clones. The Invitrogen Qubit dsDNA BR Assay Kit was used for quantification of DNA with the Invitrogen Qubit 4 Fluorometer (both supplied by Thermo Fisher Scientific Inc., Waltham, MA, USA). PCR amplifications were performed using the Phusion High-Fidelity PCR Kit (Thermo Fisher Scientific Inc., Waltham, MA, USA) or the Type-it Microsatellite PCR Kit (Qiagen GmbH, Hilden, Germany). PCR products were purified with the Roche High Pure PCR Product Purification Kit (Merck KGaA, Darmstadt, Germany). Clonings were performed using the TOPO XL-2 Complete PCR Cloning Kit (Thermo Fisher Scientific Inc., Waltham, MA, USA). The kits were used according to the manufacturer’s recommendations, and the specifications are given below.

### 4.2. Guide RNA Design and Determination of DNA Cleavage Efficiency

Guide RNAs for *Lb*Cas12a targeting exons two, five, and seven of the *PDS* gene (MD04G1023800) from *Malus domestica* (Figure 1A) were designed using CRISPR RGEN Tools (Cas-designer, Cas-OFFinder, http://www.rgenome.net/, accessed on 12 September 2019 [52,53]). The PAM type 5’-TTTV-3’ and the *Malus domestica* genome v3.0 from the genome Database for Rosaceae (GDR, https://www.rosaceae.org/, accessed on 12 September 2019) were used as parameters for crRNA design. The selected crRNAs contain a 23 bp target specific sequence (Table S1) and were ordered as HPLC purified RNA oligonucleotides. The cleavage efficiency of the crRNAs was determined by in vitro DNA cleavage assays and performed as follows. DNA fragments containing the target sequence were amplified by PCR (Figure 1C) from genomic DNA of the apple *M. domestica* cultivars ‘Pinova’, ‘Golden Delicious’, ‘Braeburn’, and ‘Gala’, using the Phusion High-Fidelity PCR Kit with 1 µM of each primer, 50 ng template DNA, 1x HF buffer in a final volume of 50 µL and 30 cycles. The annealing temperature (T_a_) and elongation time (E) of the PCR program was set specifically for each primer pair (EX1-FW/EX6-REV: T_a_ 65 °C, E 90 s; EX6-FW/EX8-REV: T_a_ 57 °C, E 60 s). The PCR products were purified, eluted with elution buffer, and quantified using the NanoDrop 2000c (Thermo Fisher Scientific Inc., Waltham, MA, USA). In vitro DNA cleavage assays were performed in a reaction volume of 30 µL using a final concentration of 30 nM EnGen LbaCas12a (Cpf1) (M0653S, New England Biolabs GmbH, Frankfurt am Main, Germany). Cas12a was mixed with 30 nM crRNA in 1× NEB Buffer 2.1. After pre-assembly for 10 min at 25 °C, the respective PCR product (3 nM) was added and the reaction was incubated at 37 °C for 10 min. The reaction was stopped by addition of 1 µL Proteinase K (New England Biolabs GmbH, Frankfurt am Main, Germany) and the resulting DNA fragments were analyzed by gel electrophoresis using 1% TAE agarose gel.

### 4.3. Transformation of the CRISPR/LbCas12a Construct

The binary vector p9oN-U10LbCpf1-Ex27 (Figure 2A) was assembled by a DNA cloning company (DNA Cloning Service, Hamburg, Germany) and transformed into the *Agrobacterium tumefaciens* strain EHA105 [54]. A proliferating in vitro shoot culture of the cultivar ‘Gala’ was used for T-DNA transformation, which was done according to the protocol described by Flachowsky et al. [55]. Regenerated meristems (so-called regenerates) were excised from the callus material, sub-cultured on shoot proliferation medium, grown in a culture chamber (day: 16 h of light at 21 °C, night: 8 h of dark at 16 °C), and propagated every four to six weeks. The selective agent kanamycin (100 mg/L) was supplemented to regeneration and shoot proliferation media for selection of transgenic plant material.

### 4.4. Detection of Transgenic Elements by PCR

Genomic DNA from whole in vitro shoots exhibiting the albino phenotype (Figure 2B) was extracted, quantified using the Qubit 4 Fluorometer, and the DNA concentration was adjusted to 10 ng/µL in ddH_2_O. The PCR analyses were performed in a final volume of 10 µL using the Type-it Microsatellite PCR Kit adding the Q-solution and 10 ng template DNA. The presence of the *LbCas12a* gene was tested using the primer pair Cas12a-FW/REV (95 °C for 3 min; 36 cycles of 95 °C for 3 min; 65 °C for 1 min; 72 °C for 1 min; 72 °C for 4 min) and the amplification of the *nptII* resistance cassette was performed with nptIIopt_F/R (95 °C for 3 min; 36 cycles of 95 °C for 30 s; 62 °C for 1 min; 72 °C for 90 s; 72 °C for 5 min). For control, the endogenous gene *MdEF1α* coding for elongation factor 1α [56] was amplified from the same template DNA using EF1a-FW/REV (94 °C for 2 min; 36 cycles of 94 °C for 30 s; 56 °C for 1 min; 72 °C for 90 s; 72 °C for 10 min). PCR products were analyzed by gel electrophoresis using 1% TAE agarose gel.

### 4.5. Genotyping of Indels by Fluorescent PCR Capillary Gel Electrophoresis

The *MdPDS* target loci A and D were amplified in a PCR multiplex with fluorescent labeled primers (Figure 1B, Table S1) using the Type-it Microsatellite PCR Kit in a final volume of 10 µL and 10 ng template DNA. The primer A-FW was 5’-labeled with the blue fluorescent dye 6-FAM and used in combination with A-REV to amplify locus A. The amplification of locus D was performed with primer D-FW together with D-REV, which was 5’-labeled with the green fluorescent dye Atto532. The PCR was performed with the following conditions: initial denaturation at 95 °C for 5 min, 30 cycles of 95 °C for 30 s, 60 °C for 90 s, 72 °C for 1 min, and a final elongation step at 60 °C for 30 min. Before, the annealing temperature was optimized by gradient PCR. For fragment length analysis of the PCR products, the PCR reaction was diluted to the ratio 1:100 with ddH_2_O. One µL of the diluted PCR reaction was supplemented with 8.95 µL Applied Biosystems Hi-Di Formamide and 0.05 µL Applied Biosystems GeneScan 600 LIZ dye Size Standard v2.0 (both supplied by Thermo Fisher Scientific Inc., Waltham, MA, USA). After denaturation at 95 °C for 5 min, capillary gel electrophoresis was performed on the Applied Biosystems Genetic Analyzer 3500xl system (Thermo Fisher Scientific Inc., Waltham, MA, USA) using the POP-7 Polymer (Thermo Fisher Scientific Inc., Waltham, MA, USA). The fragment length analysis was done with the Applied Biosystems GeneMapper Software 6.

### 4.6. Amplicon Deep Sequencing of MdPDS Target Sites

PCR products intended for sequencing were amplified with the Phusion High-Fidelity Polymerase Kit using 1 µM of each primer (without fluorescent label), 10 ng template DNA, 1× HF buffer in a total volume of 50 µL, and 30 cycles. Using these conditions, the annealing temperature was optimized for each primer pair by gradient PCR (69 °C for A_FW/REV; 60 °C for D_FW/REV). After purification of the PCR products, the DNA concentration was determined on the Qubit 4 Fluorometer. Library preparation and amplicon deep sequencing on the Illumina platform with a 2 × 250 bp configuration was performed by Genewiz Germany GmbH (Leipzig, Germany). The subsequent analysis of the sequencing data was done on the Galaxy platform [57] with a created workflow. First, merging of the paired-end reads was performed with USEARCH (Galaxy Version 1.0.0). After, the reads were converted to FASTA sequences using the FASTQ-to-FASTA converter (Galaxy Version 1.1.1). The abundance of unique sequences was determined with VSearch dereplication (Galaxy Version 2.8.3.0) using both strands for clustering. The output file was created by the FASTA-to-Tabular converter (Galaxy Version 1.1.0). Sequences with an abundance ≥1% were considered for further statistical analysis. An alignment of all sequences to the wt reference sequence from ‘Gala’ was created with the CLC Main Workbench 21 (Version 21.0.1) to characterize the type of GE event and the length of insertions or deletions at the target site. The percentage of reads of sequences with identical GE events was calculated.

### 4.7. Cloning and Sanger Sequencing

The chromosomal fragment, including both *MdPDS* target loci (4.3 kb), was amplified with the primer pair A_FW/D_REV using the Phusion High-Fidelity PCR Kit (30 cycles of 95 °C for 10 s; 65 °C for 1 min; 72 °C for 2 min 15 s; and a final elongation step at 72 °C for 5 min). The PCR product was cloned into pCR-XL-2-TOPO using the TOPO XL-2 Complete PCR Cloning Kit and transformed into the One Shot OmniMAX 2 T1 chemically competent cells included in the cloning kit. Bacterial clones were selected on the LB medium containing kanamycin and analyzed for the presence of the *MdPDS* specific fragment by colony PCR (primer pairs: M13-FW/REV; A_FW/REV; D_FW/REV). Sanger sequencing of plasmid DNA isolated from PCR positive clones was done by Eurofins Genomics Germany GmbH (Ebersberg, Germany).

## Figures and Tables

**Table 1:** Comparison of results obtained by amplicon deep sequencing and fluorescent PCR capillary gel electrophoresis.

Material		Locus A									Locus D								
		Deep Sequencing				Fragment Length Analysis					Deep Sequencing				Fragment Length Analysis				
Regenerate	Shoot	Reads analyzed	Type	Diff. from wt	[%] Reads	Allele size	Diff. to wt	Peak Height	Sum Peak Heights	[%] Peak Height	Reads analyzed	Type	Diff. from wt	[%] Reads	Allele size	Diff. from wt	Peak Height	Sum Peak Heights	[%] Peak Height
Gala		163,807	wt	0	100.0	430	0	13,617	13,617	100.0	151,045	wt	0	100.0	377	0	21,545	21,545	100.0
18 219	a	197,114	Del	−84	62.9	346	−84	11,069	18,988	58.3	167,120	Del	−12	51.0	365	−12	13,582	26,958	50.4
			wt	0	35.1	430	0	7480		39.4		Del	−2	49.0	375	−2	13,376		49.6
			Del	−4	2.1	426	−4	439		2.3									
18 234	1	215,296	Del	−1	38.6	429	−1	6854	17,093	40.1	136,560	wt	0	43.8	377	0	9734	23,160	42.0
			Del	−4	33.4	426	−4	5267		30.8		Del	−8	27.8	369	−8	6586		28.4
			wt	0	25.6	430	0	4552		26.6		Del	−10	16.2	367	−10	3769		16.3
			Del	−8	2.4	422	−8	420		2.5		Del	−13	7.2	364	−13	1657		7.2
												Del	−17	1.9	360	−17	499		2.2
												Del	−16	1.7	361	−16	406		1.8
												Del	−9	1.4	n.d.				
															358	−19	509		2.2
18 238	a	202,366	Del ^a^	−4	80.4	426	−4	5580	6604	84.5	125,784	wt	0	79.9	377	0	7954	9010	88.3
			wt	0	14.8	430	0	1024		15.5		Del	−16	11.6	361	−16	1056		11.7
			Del	−6	2.8	n.d.						Del ^g^	−6	3.6	n.d.				
			Del ^b^	−5	1.9	n.d.						Del	−9	2.7	n.d.				
												Del ^h^	−13	2.2	n.d.				
	b	183,884	Del ^a^	−4	78.2	426	−4	11,714	15,395	76.1	143,519	Del ^i^	−18	23.1	359	−18	4320	19,405	22.3
			wt	0	19.1	430	0	3149		20.5		Del ^k^	−12	22.4	365	−12	4453		22.9
			Del	−10	2.7	420	−10	532		3.5		wt	0	19.5	377	0	3917		20.2
												Del	−14	16.8	363	−14	3507		18.1
												Del	−20	16.2	357	−20	3208		16.5
												Del ^h^	−13	2.0	n.d.				
	c	172,597	Del ^b^	−5	40.4	425	−5	1545	3904	39.6	155,911	Del ^k^	−12	39.8	365	−12	3956	5,335	74.2
			Del ^a^	−4	37.2	426	−4	1300		33.3		Del	−12	38.9					
			wt	0	22.4	430	0	1059		27.1		wt	0	19.5	377	0	910		17.1
												Del	−8	1.8	369	−8	150		2.8
															370	−7	173		3.2
															371	−6	146		2.7
	d	192,845	Del ^a^	−4	63.2	426	−4	6797	10,654	63.8	126,005	Del	−14	44.8	363	−14	5807	13,844	41.9
			wt	0	36.8	430	0	3857		36.2		Del	−4	30.6	373	−4	4289		31.0
												Del ^i^	−18	13.2	359	−18	1857		13.4
												wt	0	7.7	377	0	1010		7.3
												Del	−19	2.0	358	−19	500		3.6
												Del ^g^	−6	1.7	371	−6	381		2.8
18 268		201,374	wt	0	93.6	430	0	15,210	16,634	91.4	207,734	wt	0	92.2	377	0	23,847	26,738	89.2
			Del	−10	4.2	420	−10	919		5.5		Del	−38	2.2	339	−38	743		2.8
			Del	−14	2.2	416	−14	505		3.0		Del	−15	2.0	362	−15	754		2.8
												Del	−5	1.8	372	−5	737		2.8
												Del	−11	1.8	366	−11	657		2.5
18 298	c	201,668	Del ^c^	−12	43.6	418	−12	7547	14,362	52.5	182,157	Del ^m^	−20	50.4	357	−20	10,278	20,324	50.6
			wt	0	37.2	430	0	6815		47.5		Del ^o^	−9	49.6	368	−9	10,046		49.4
			Art ^e^	−165	17.0	n.d.													
			Art ^f^	−172	2.3	n.d.													
	d	204,297	Del ^c^	−12	52.0	418	−12	9693	18,744	51.7	182,047	Del ^m^	−20	51.6	357	−20	12,387	24,866	49.8
			Del ^d^	−4	48.0	426	−4	9051		48.3		Del ^o^	−9	48.4	368	−9	12,479		50.2
	e	210,743	Del ^c^	−12	50.8	418	−12	4822	9280	52.0	188,583	Del ^m^	−20	52.7	357	−20	7393	14,335	51.6
			wt	wt	28.5	430	0	3066		33.0		Del ^o^	−9	47.3	368	−9	6942		48.4
			Del	−7	10.3	423	−7	1392		15.0									
			Art ^e^	−165	8.2	n.d.													
			Art ^f^	−172	2.3	n.d.													
	i	221,032	Del ^c^	−12	49.7	418	−12	9484	19,358	49.0	197,422	Del ^m^	−20	48.9	357	−20	13,184	27,323	48.3
			wt	0	45.1	430	0	9175		47.4		Del ^o^	−9	46.2	368	−9	12,774		46.8
			Del ^d^	−4	3.1	426	−4	699		3.6		wt	0	4.9	377	0	1365		5.0
			Art ^e^	−165	2.0	n.d.													

In vitro shoots with the albino phenotype were analyzed as described in detail in Figure 3. The size difference from the wt sequence was calculated for each sequence/fragment and this parameter was used to assign potentially identical GE events detected by both methods. GE events characterized by sequencing that were not detected by fragment length analysis are marked with n.d. Identical GE events with the same unique sequence in different shoots of the same regenerate are indicated by the superscript letters (^a, b, c, d, e, f, g, h, i, k, m, o^). The peak height (intensity of fluorescence (RFU)) of each fragment is given. The sum of peak heights was used to calculate the percentage of total peak height for each fragment. (wt: wild type sequence; Del: deletion within target site; Art: potential artefacts).

## Data Availability

The data presented in this study are available in the article and the Supplementary Materials here.

## Supplementary Materials

The following are available online at https://www.mdpi.com/article/10.3390/ijms222212611/s1.
