# Supplementary material for: Tracing CRISPR/Cas12a Mediated Genome Editing Events in Apple Using High-Throughput Genotyping by PCR Capillary Gel Electrophoresis
Source: Int J Mol Sci. 2021 Nov 22;22(22):12611. doi: 10.3390/ijms222212611 (PMC8619667; doi:10.3390/ijms222212611)
Supplement: Supplementary file 1 [file ijms-22-12611-s001.zip › ijms-1443841-supplementary.pdf]

International Journal of *Molecular Sciences*

MDPI

Supplementary Material

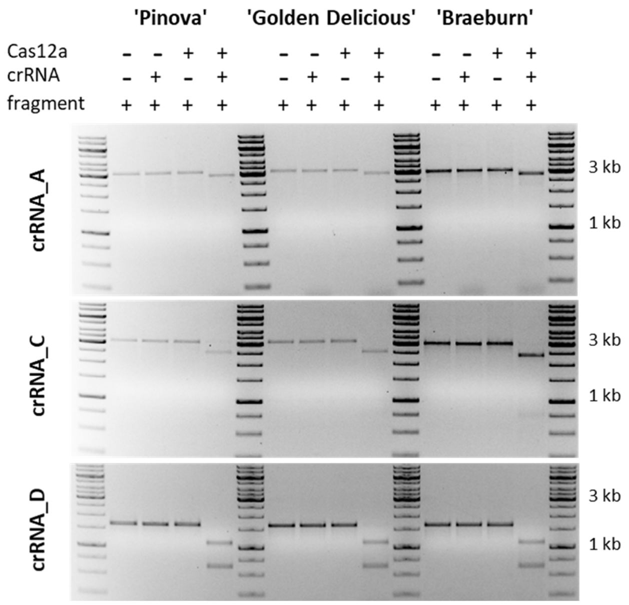

Figure S1. Analysis of cleavage efficiency of crRNAs on different apple genotypes. PCR fragments amplified from different apple cultivars ('Pinova', 'Golden Delicious', 'Braeburn') were used as substrate for *in vitro* cleavage reactions in combination with different crRNAs pre-assembled with *Lb*Cas12a (Figure 1). The use of crRNA_A, C and D resulted in full digestion of the DNA substrate of each genotype.

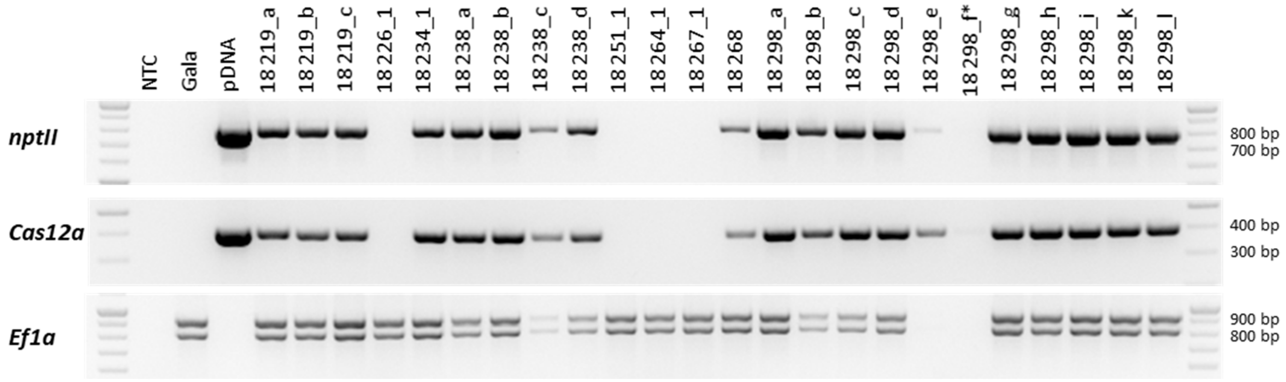

Figure S2. Detection of transgenic *nptII* and *LbCas12a* sequences in albino shoots. PCR using *nptII* and *LbCas12a* specific primer pair were performed to analyze several albino shoots. The plasmid p9oN-U10LbCpf1-Ex27 (pDNA), the genomic DNA of 'Gala' as well as no template control (NTC) were performed as PCR controls. Using a universal primer pair amplifying *EF1α* sequences, each DNA sample was tested for their usability as PCR template.

Table S1: DNA and RNA oligonucleotides. The sequence of the oligonucleotides used as crRNA or DNA primer for PCR amplification, respectively, is given. The 23 bp target-specific sequence of the respective crRNA is underlined. Fluorescent 5'-labels of the PCR primers are indicated.

oligo name	5'-label	5'-sequence-3'
**guide RNA:**		
crRNA_A		UAA UUU CUA CUA AGU GUA GAU CGA GCC UCU CCU CGU CCA ACC AA
crRNA_C		UAA UUU CUA CUA AGU GUA GAU GCA UUG CAA AUA UCA UAG AAU GU
crRNA_D		UAA UUU CUA CUA AGU GUA GAU UAG CCA UGU CAA AGG CCC UUA AC

**PCR primer:**		
EX1-FW		AGG AAT GGT GTT TGC CCC TT
EX6-REV		AAC ATA AGC CTG CCC ACC AA
EX6-FW		TGT TGA AGC CCA AGA TGG CT
EX8-REV		GTG GCG AAC ACA TAC GCA TC
A-FW		TGG CAT AGG TTG TTT GCG TTG
A-FW	6-FAM	TGG CAT AGG TTG TTT GCG TTG
A-REV		TAA AGT TAG AGC GGC CGA AAA C
D-FW		TTT TGG TGA TTC GCA GGC CA
D-REV		GGT GCA CAT GAA GCC ATA ACA T
D-REV	Atto532	GGT GCA CAT GAA GCC ATA ACA T
EF1a-FW		ATT GTG GTC ATT GGY CAY GT
EF1a-REV		CCA ATC TTG TAV ACA TCC TG
nptIIopt_F		AGC ATG GAT TGA GCA GGA
nptIIopt_R		ATG ATG TTG GGG AGG CAG
Cas12a-FW		CCA GTA TCG CTT TTC GCT GC
Cas12a-REV		CTC CTC GTC GGA TGT GTA GC
